# Visual fields in neuro-ophthalmology

**DOI:** 10.4103/0301-4738.77013

**Published:** 2011

**Authors:** Sachin Kedar, Deepta Ghate, James J Corbett

**Affiliations:** Department of Neurology, University of Kentucky College of Medicine, Lexington, KY; 1 Department of Neurology, University of Mississippi Medical Center, Jackson, MS, USA

**Keywords:** Hemianopia, ischemic optic neuropathy, optic chiasm, optic neuritis, perimetry, visual fields

## Abstract

Visual field assessment is important in the evaluation of lesions involving the visual pathways and should be performed at baseline and periodically in the follow-up. Standard automated perimetry has been shown to be adequate in neuro-ophthalmic practise and is now the technique of choice for a majority of practitioners. Goldman kinetic visual fields are useful for patients with severe visual and neurologic deficits and patients with peripheral visual field defects. Visual fields are useful in monitoring progression or recurrence of disease and guide treatment for conditions such as idiopathic intracranial hypertension (IIH), optic neuropathy from multiple sclerosis, pituitary adenomas, and other sellar lesions. They are used as screening tools for toxic optic neuropathy from medications such as ethambutol and vigabatrin. Visual field defects can adversely affect activities of daily living such as personal hygiene, reading, and driving and should be taken into consideration when planning rehabilitation strategies. Visual field testing must be performed in all patients with lesions of the visual pathway.

A detailed neuro-ophthalmic assessment of a patient with visual symptoms should include visual field testing. While there are clear guidelines for the performance and interpretation of visual fields in glaucoma, very few exist for neuro-ophthalmic disorders. Visual fields serve three important purposes in neuro-ophthalmology.

1. *Diagnostic*: Visual field defects indicate involvement of the visual pathways and the pattern of visual field defects help in localizing site of the lesion.
2. *Follow-up*: Visual fields provide an excellent tool to monitor resolution and/or recurrence of disease processes affecting the visual pathways.
3. *Activities of daily living*: Since visual field defects adversely affect the patient’s ability to perform day-to-day activities such as personal hygiene, reading, and driving, these defects should be actively sought when planning rehabilitation strategies.

We reviewed the current English language medical literature on Medline using the following search words: visual field defects, papilledema, idiopathic intracranial hypertension (IIH), optic neuritis, optic neuropathy, ischemic optic neuropathy, optic chiasm, and hemianopia. Through this review, we attempt to summarize the role of visual fields in neuro-ophthalmic conditions.

## Visual Field Techniques

Visual field testing can be performed by a number of different techniques including confrontation (at the bedside), tangent screen, Goldmann kinetic perimetry, and automated static perimetry. The sensitivity of confrontation techniques is about 20% for detection of arcuate field defects and about 70% for detection of hemianopia when compared to Goldmann[1] and automated perimetry.[2] The use of a small 5-mm red target in a kinetic and static manner seems to have the best sensitivity (about 75%) for detecting a variety of visual field defects,[3] especially when combined with the red color comparison technique. Confrontation techniques have a high specificity (97%) and positive predictive value (96%)[4] for detection of visual field defects when confirmed with standard automated perimetry (SAP).

SAP has replaced Goldmann perimetry in clinical practice amidst fears that peripheral visual field defects may be missed. This fear seems to be unwarranted as only 1–2% patients with nonglaucomatous visual field defects have abnormalities in the peripheral field beyond 30° in the absence of a central field defect.[5] Patients with severe vision loss (visual acuity worse than 20/200) or severe neurologic deficits (modified Rankin score more than three) preferred Goldmann perimetry to SAP using SITA Fast strategy. In both these patient groups, automated perimetry and Goldmann perimetry were found to be reliable and reproducible in 75% of the patients.[6] Swedish interative thresholding algorithm (SITA) fast visual field strategy may also offer a reliable alternative to Goldmann perimetry in children.[7]

Frequency doubling technology (FDT) perimetry, which was developed as a screening test for glaucoma, was found to have a similar sensitivity and specificity to SAP in patients with optic neuropathy,[8] but not in patients with hemianopia due to an inability to define the vertical limit of these defects.[9] FDT perimetry seems unable to accurately differentiate and categorize hemianopic, quadrantranopic, or glaucomatous field defects.[10] However, the second generation FDT (Humphrey Matrix) was found to have fair-to-good concordance with SAP for optic nerve and chiasmal disorders[11] and a marginally lesser sensitivity in detecting homonymous hemianopia.[12]

**Figure 1:**
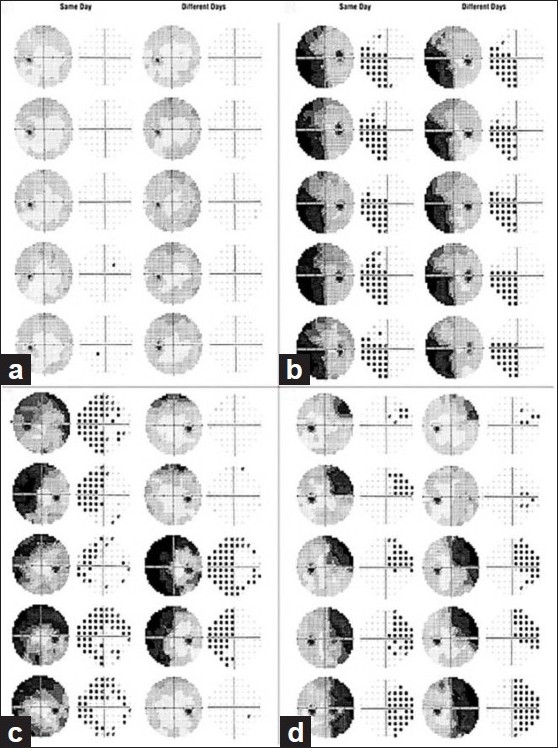
Gray-scale and probability results in healthy subjects and patients with optic neuritis. Serial visual fields were performed either on the same day or different days. The two fields at the top of each graph are from 8 AM, the next pair down from 10 AM, etc. (a) Typical example of a normal subject’s consistent results. (b) A patient with optic neuritis with consistent results. (c, d) Variable results in patients with optic neuritis. Note the variation from near normal to near complete hemianopia (Reproduced with permission of the American Medical Association. From Wall *et al*.[14] Copyright © 1998. American Medical Association. All rights reserved.)

SAP can be used to monitor disease processes by following changes in visual field defects. However, it may not be easy to differentiate true changes from long-term and short-term variabilities in the threshold measurements, especially in the areas of visual field damage. In a study of the follow-up visual fields of patients enrolled into the ocular hypertension treatment study (OHTS), 86% of patients with visual field abnormalities were found to have normal visual fields on subsequent examinations. All patients in the OHTS trial had normal and reliable visual fields at enrollment.[13] Patients with recovered optic neuritis also showed significant variability of the threshold measurements between consecutive tests performed on the same day as well as different days as compared to normal age-matched controls[14] and patients with glaucoma [Fig. 1].

In summary, confrontation visual fields are useful at the bedside when combinations of techniques are used. SAP provides adequate testing of the visual field in a majority of neuro-ophthalmic patients. Goldmann perimetry is useful in patients with severe visual and neurological deficits or patients with “isolated peripheral visual field defects”. However, lack of trained technicians limits the use of Goldmann perimetry to a few centers.

## Papilledema and IIH

**Figure 2:**
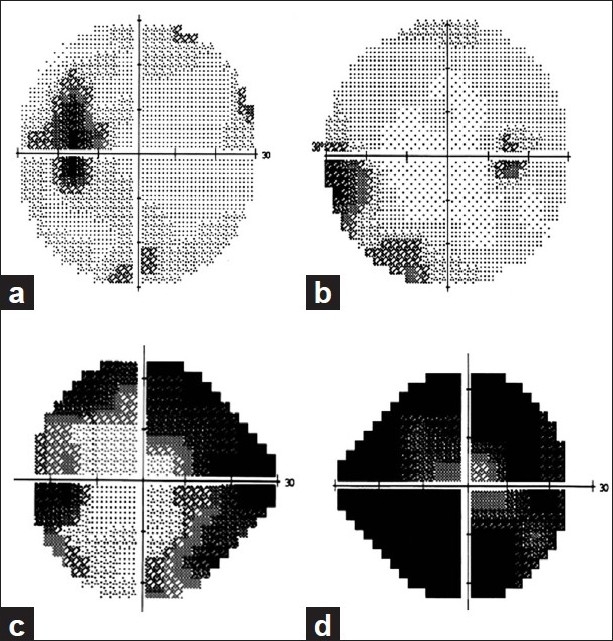
Visual field defects in idiopathic intracranial hypertension. (a) Enlarged blind spot. (b) Nasal step. (c) Biarcuate scotoma. (d) Severe visual field constriction

Papilledema refers to the swelling of the optic nerve head secondary to raised cerebrospinal fluid (CSF) pressure. IIH is defined as raised intracranial pressure (ICP) in the absence of radiologic and laboratory abnormalities reflecting any other known cause of raised ICP.[15] Vision loss is the most feared complication of IIH with at least 10% of patients progressing to blindness from IIH.[16] The visual field defects that result from papilledema in IIH are “disc-related defects” and are similar to those found in glaucoma.[17] Visual field losses may be identified in as many as 96% of patients using disease-specific perimetric strategies such as the Armaly-Drance strategy for Goldmann perimetry or SAP. The most common defects seen in IIH are blind spot enlargement, generalized constriction, and loss of the nasal visual fields, especially inferonasal [Fig. 2].[18] Other common field defects described include inferior altitudinal loss, superonasal and superotemporal loss,[19] arcuate defects, and scotomas (central, cecocentral, and paracentral).[20] The commonly used visual field strategies such as full threshold and SITA standard were found to be comparable in the detection of visual field loss on individual examinations.[21] Patients with IIH should be followed with sequential quantitative perimetry to aid rational decision making and prevent vision loss. The same visual field testing strategy should be used at each visit to obtain comparable data in follow-up.

## Optic Neuritis and Multiple Sclerosis

The optic neuritis treatment trial (ONTT) has provided valuable information about the natural history of optic neuritis and the response to treatment with corticosteroids. In the ONTT, visual field administration was standardized to include central field testing (Humphrey Visual Field Analyzer full-threshold strategy of the central 30-2) as well as two peripheral isopters on the Goldmann kinetic perimeter. All visual field interpretations were performed at a visual field reading center.[22] The affected as well as the fellow eye was tested at initial and follow-up visits. At baseline, 100% of the affected eyes and 69% of the fellow eye showed visual field abnormalities. Visual field defects in the affected eye at presentation included diffuse visual field loss (48%), altitudinal defects (15%), central or cecocentral scotoma (8.3%), arcuate or double arcuate (4.5%), hemianopic defects (4.2%), and others.[23] Patients with hemianopic field defects (13% during the first year) were more likely to show abnormalities on the brain magnetic resonance imaging (MRI) at baseline as compared to patients without these field defects, indicating the presence of demyelinating lesions and multiple sclerosis. Follow-up visual fields of the affected eye were normal in 51% at 6 months and 56% at 1 year.[24] Fellow eye visual field defects were seen in 33% at 6 months and 31% at 1 year, of which 87% had abnormal visual fields at baseline. A mean deviation cutpoint of ≤–15 dB at the 1-month follow-up correlated with moderate-to-severe visual loss (acuity, contrast, and field) at 6 months.[25]

Between years 1 and 15 approximately 50% of the affected eyes and 34–39% of fellow eyes had abnormal visual fields.[26] Abnormal visual fields were seen in 50.6% of the affected eyes and 35.6% of the fellow eye at the 15 year follow-up which was completed by 65% of the original cohort. At final follow-up, patients who developed multiple sclerosis were more likely to have abnormal visual functions including visual fields.[27]

Irrespective of the type of the initial field defect in the affected eye, patients had recovered at least 85% of the average threshold at 6 months with recovery being maximum closest to fixation.[28] A comparison of the visual field data obtained by SAP and the kinetic perimeter showed that the peripheral visual field defects were less severe and recovered more rapidly as compared to the central visual field defect. While assessment of the central visual field by SAP is sufficient in most cases of optic neuritis, kinetic perimetry of the periphery may be obtained at baseline and followed up if the central field is severely affected.[29]

In the ONTT, visual fields were obtained at baseline and at seven follow-up visits in 6 months and then yearly. We obtain visual fields at baseline and follow-up visits at 2 weeks, 1 month, 6 months, 1 year, and annually thereafter. Visual fields help quantify the depth of visual field loss, identify atypical cases of optic neuritis, and aid in counselling patients about prognosis.

## Toxic Optic Neuropathy

### Ethambutol

Optic neuropathy from ethambutol is dependant on the dose and duration of treatment.[30] Despite the widespread use of this medication in treatment of tuberculosis, there are no clear guidelines for monitoring visual functions.[31] Screening remains the mainstay for early detection of visual field defects and is important in preventing this condition. Central scotoma is the commonest field defect seen in ethambutol optic neuropathy. Other less common field defects include peripheral isopter constriction,[32] altitudinal field defects, and rarely bitemporal field defects.[33] The onset and course of the disease are highly variable. In a prospective study of 52 patients (104 eyes) treated with ethambutol for 2 months, Menon *et al*.[34] detected visual field defects in eight eyes (7.7%) of four patients at 2 months in the form of peripheral isopter constriction on Goldmann visual fields. None of the patients had any subjective visual complaints or abnormalities of central vision as tested on the Amsler grids. Four eyes of three patients had persistent field defects 1 month after discontinuing the medication. In a retrospective study of 13 patients with ethambutol optic neuropathy, the authors noted improvement in visual fields (defined as improvement of at least 5°), in 78% of patients.[35]

Patients on ethambutol should have visual acuity, color vision, contrast sensitivity, and visual fields tested at baseline and at monthly intervals till the medication is discontinued. If visual field defects are seen, ethambutol should be discontinued and follow-up fields should be obtained every 1–3 months till visual fields have either improved or stabilized.

### Vigabatrin

Vigabatrin (VGB) is an antiepileptic medication used in the treatment of infantile spasms (IS) and refractory complex partial seizures. Visual field defects are known complications of treatment with VGB. The prevalence of the visual field defect varies with patient age: 15–31% in infants, 15% in children, and 25–50% for adults. The visual field defect usually starts as a bilateral nasal defect and progresses to concentric bilateral field defects with preservation of central vision. The time to onset of the field defect varies with patient age: 9 months (mean 4.8 years of VGB exposure) in adults; 11 months (mean 5.5 years of VGB exposure) in children, and 3.1 months to the earliest onset of VGB retinal defects in infants (defined as two consecutive electroretinogram abnormalities). Guidelines have been established for monitoring visual fields in patients being treated with this medication.[36]

Cognitive and age-appropriate visual field testing must be performed at baseline and at periodic intervals in patients who continue treatment with VGB. It is recommended for infants and children to be tested by electroretinography (ERG); static and kinetic perimetry can be used for patients 9-12 years and above.

Infants are tested at baseline and at 3-month intervals for 18 months and 6-month intervals thereafter; all others are tested at baseline and at 6-month intervals. Testing should be repeated within a month of an abnormal visual field for confirmation.

A trial of VGB can be safely performed for 12 weeks since there is only a minimal risk of visual field defects during the early phase of treatment. VGB should be discontinued if no clinical benefit is noted by 12 weeks.

## Ischemic Optic Neuropathy

Visual field defects in nonarteritic anterior ischemic optic neuropathy (NA-AION) include altitudinal field defect (classically occurring in the inferior hemifield), central scotoma, arcuate scotoma, and quadrantic defects. Hayreh and Zimmerman[37] systematically reviewed Goldmann visual fields in their large series of 312 patients with NA-AION and found that an absolute inferior nasal sector defect was the most common type of visual field defect using a V-4e isopter. Diffuse central field defects are more commonly seen with SAP.[38] Similar results were seen in the ischemic optic neuropathy decompression trial (IONDT). While a superior or inferior arcuate defect was the commonest defect, an additional central scotoma was present in eyes with visual acuity worse than 20/64 at baseline.[39] Visual field defects have also been noted in the “spared hemifields” of patients with AION.[40]

A follow-up study of 332 patients with NA-AION (minimum follow-up period of 8 weeks) showed that those who presented within 2 weeks of symptom-onset had a greater likelihood of visual field changes (either improvement or worsening) compared to those who presented after 2 weeks.[41] This suggests that the maximum visual field change occurs within the first few weeks. No significant change in the visual field was noted after a 6-month follow-up period. Overall, central visual fields improved in 16% and worsened in 16% of eyes while peripheral fields improved in 17% and worsened in 18%. Similar results were seen in the follow-up studies of patients in the IONDT.[42]

In patients with posterior ischemic optic neuropathy (PION), central visual field loss was also the most common type of field defect. Steroids were found to have a beneficial effect on the visual fields of both arteritic and nonarteritic PION.[43]

In patients with NA-AION, visual fields should be obtained at baseline and every 2 weeks until there is resolution of disc edema and then monthly till the fields stabilize. It is important to test both central and peripheral fields.

### Hereditary optic neuropathy

Hereditary optic neuropathies cause progressive deterioration of vision. Visual field examination is useful in monitoring the progress of the condition. By quantifying visual loss, patients can be counseled about lifestyle modifications and use of low vision aids.

Leber’s hereditary optic neuropathy (LHON) is a maternally-inherited optic neuropathy caused by mutations of the mitochondrial DNA. It affects central vision with progressive deterioration of visual acuity and fields. Central or cecocentral scotomas are the classic visual field defects in LHON. The natural history of visual field defects in LHON was studied in the unaffected fellow eyes of nine patients with genetically proven LHON.[44] A majority of the fellow asymptomatic eyes of patient with LHON showed minimal changes on the pattern deviation plots in the paracentral visual fields despite normal mean deviations (MD) at baseline. These defects progressed rapidly with worsening of MD and foveal sensitivity at 1-month follow-up to become central scotomas in a pattern similar to the fellow eye. Studies have also shown visual field defects in the asymptomatic maternal relatives of patients with LHON.[45,46]

Patients with dominant optic atrophy (DOA) demonstrate a spectrum of centrocecal field defects of variable degrees with very few patients showing peripheral visual field defects.[47] There seems to be a significant correlation between the severity of the visual field defect (mean deviation on SAP) and the duration of the disease.[48] The field defects seem to be significantly worse when tested with short wavelength automated perimetry (SWAP) where the MD were depressed 10–20 times more than MD in SAP.[49]

SAP is useful in following up visual field defects in patients with hereditary optic neuropathy and should be performed at baseline and periodically, based on the clinical presentation. SWAP can detect visual field changes in the very early stages of the disease.

### Chiasmatic disorders

Lesions of the optic chiasm can produce a variety of visual field defects including bitemporal hemianopia, junctional scotoma (anterior chiasmal defect), quadrantanopia and bitemporal, or unilateral temporal scotoma depending on the site and extent of the lesion. SAP can show temporal depression and vertical step very early in chiasmal lesions even with normal Goldmann fields.[50]

Visual field improvement following resection of the pituitary tumor occurs in three stages.[51] Stage one is the early fast phase of recovery seen within few days to a week of the surgery. In a few individuals, there can be complete normalization of the visual fields. Stage two is the phase of slow recovery which is seen within a few weeks of the surgery to a few months. During this stage, the visual fields show significant and presumably slow and sustained improvement.[52] Stage three is the late phase starting a few months after decompression to a few years. During this stage, there is minimal improvement of the visual fields. Some studies have identified improvement as long as 5 years following surgical resection.[52] Poor prognostic signs for improvement of visual fields include dense and extensive preoperative visual field deficit,[52] pituitary tumor volume greater than 5 cc[53] and the postoperative development of a surgically “empty sella”[54] (which is associated with inflammatory scarring and descent of the chiasm into the empty sella).

Visual fields should be obtained periodically (1–3 months or more frequently) based on the clinical presentation, lesion characteristics, type of intervention (surgical, medical, or radiation), and patient’s visual complaints. SAP seems to be more sensitive in detecting early field defects. Goldmann fields may be used to assess peripheral fields in patients with advanced field defects. Visual fields are crucial in guiding ongoing treatment and judging treatment success in a number of patients with sellar lesions.

### Retrochiasmal disorders

Retrochiasmal disorders produce varied patterns of homonymous hemianopia, depending on the site of the lesion. Before the advent of modern neuroimaging, these patterns were used to localize a lesion in the visual pathway. However, a recent study has shown that the visual field defects may not be as specific to a given location as was previously believed.[55] Lesions anywhere along the retrochiasmal pathway can produce virtually any type of homonymous visual field deficit, except a unilateral loss of the temporal crescent and homonymous sectoranopia that are produced exclusively by anterior occipital and geniculate lesions, respectively. Contrary to existing and persistent belief, almost 50% of lesions involving the optic tracts produce congruent homonymous hemianopia even though they have a reputation for being notoriously incongruous.[56] Congruent homonymous hemianopias are produced by posterior pathway lesions and the chance that a congruent homonymous hemianopia is produced by a lesion involving the occipital lobe was estimated to be 56%.

In a recent study on the natural history of homonymous hemianopia, spontaneous improvement of the visual field defect has been observed in 38% of patients.[57] Visual field improvement was defined as an improvement of the field defect by at least 10° horizontally and 15° vertically using similar isopters on the Goldmann visual fields and significant changes in mean and pattern deviations in Humphrey visual fields. The probability of finding an improvement in the visual field defects was 50–60% if the initial visual fields were obtained within a month of the neurological injury and this dropped to about 20% if the initial fields were obtained after 6 months. No other factor including age seemed to affect significantly the chances of visual field improvement.[58] The natural history of visual field improvement is especially important when evaluating claims of improvement by potential rehabilitation treatments for homonymous hemianopia.

Patients with retrochiasmal lesions can have other neurologic manifestations such as motor deficits that require rehabilitation. Visual field defects can hinder early rehabilitation of patients with other neurologic deficits such as hemiparesis. Studies have shown that in patients with hemiparesis from a unilateral hemispheric stroke, the probability of achieving relative independence in ambulation and self-care function diminished significantly when accompanied by a visual field defect.[59]

Visual fields should be assessed in all patients who present with lesions that are close to or involve the visual pathway. Follow-up visual fields may be obtained at 6- to 8-week intervals (earlier for more aggressive lesions such as tumors) until the fields have stabilized. Patients unable to meet the rehabilitation goals in a timely manner should be suspected to have additional deficits such as visual field defects and evaluated by perimetry.

## Quality of Life

Visual field deficits arising from neuro-ophthalmic conditions can adversely affect the quality of life and activities of daily living. Abnormalities on the subscales of the National Eye Institute Visual Function Questionnaire (NEI-VFQ 25)[60] are seen in different neuro-ophthalmic conditions including optic neuropathy from multiple sclerosis,[61,62] chiasmal defects from pituitary adenoma,[63] and homonymous hemianopia from retrochiasmal lesions[64] (infarct,[65,66] trauma, tumor, and hemorrhage). Deficits on the VFQ subscales often do not correlate with objective parameters of visual function and an understanding of the different subscales and how it affects certain visual tasks may aid in planning better assessment strategies and rehabilitation.[67]

**Figure 3:**
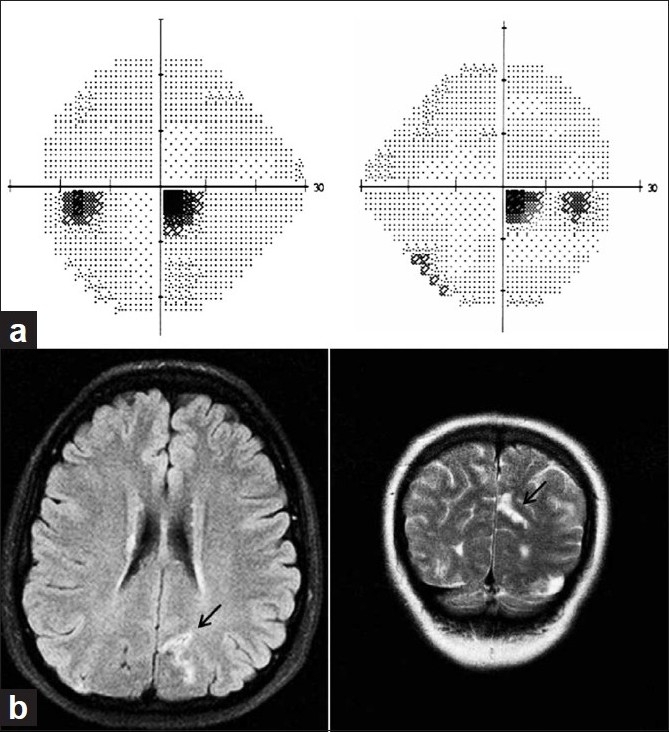
(a) Visual fields of a 38-year-old who complained of severe difficulty reading, shows a right inferior homonymous scotoma. (b) MRI brain of the same patient shows a lesion in the superior bank of the left calcarine cortex

Homonymous hemianopia causes patients to have impairment of daily activities such as personal hygiene, meal preparation, driving, shopping, and telephone usage.[68] Patients with homonymous hemianopia involving the central 5° complain of difficulty in reading, and are classified as “hemianopic dyslexia” [Fig. 3].[69] Patients with homonymous paracentral scotoma may be impaired while driving despite having a relatively large visual field intact. The scotomatous area often overlies the side-view mirror on one side and impairs the ability to change lanes safely. The subject of minimum visual field requirements for driving has been one of much debate. Hemianopic patients have demonstrated poor blind side hazard detection for pedestrians that were not compatible with driving in driving simulators.[70] Patients with hemianopia and quadrantanopia (especially inferior quadrantic defects) were noted to have difficulty with lane position/lane change, steering steadiness, and gap judgment compared to normal controls.[71] Other studies dispute the importance of visual fields for driving safety and standards[72] and suggest that assessment of on-road driving performance under the supervision of a trained specialist may be the best option. The International Council of Ophthalmology (ICO) recommends a binocular continuous field of 120° in the horizontal meridian split approximately between the right and left halves[73] as the minimum visual field requirement for driving.

## Summary

Visual fields should be obtained in all patients having lesions that either involve or are in close proximity to the visual pathways. SAP has been shown to be adequate in testing visual fields in the neuro-ophthalmic population at initial presentation as well as for monitoring progressive or recurrent conditions. Goldmann perimetry is useful in patients with advanced neurologic and/or visual deficits but is limited by the lack of trained technicians. Visual field defects have a significant impact on the quality of life and should be considered mandatory in the rehabilitation process especially if the patient can perform perimetry.
